# Biomaterial‐Based Metabolic Regulation in Regenerative Engineering

**DOI:** 10.1002/advs.201900819

**Published:** 2019-07-28

**Authors:** Chuying Ma, Michelle L. Kuzma, Xiaochun Bai, Jian Yang

**Affiliations:** ^1^ Department of Biomedical Engineering Materials Research Institute The Huck Institutes of the Life Sciences The Pennsylvania State University University Park PA 16802 USA; ^2^ Academy of Orthopedics Guangdong Province Provincial Key Laboratory of Bone and Joint Degenerative Diseases The Third Affiliated Hospital of Southern Medical University Guangzhou 510280 China; ^3^ Department of Cell Biology Key Laboratory of Mental Health of the Ministry of Education School of Basic Medical Sciences Southern Medical University Guangzhou 510515 China

**Keywords:** biomaterials, energy metabolism, metabolic regulation, metabonegenic regulation, regenerative engineering

## Abstract

Recent advances in cell metabolism studies have deepened the appreciation of the role of metabolic regulation in influencing cell behavior during differentiation, angiogenesis, and immune response in the regenerative engineering scenarios. However, the understanding of whether the intracellular metabolic pathways could be influenced by material‐derived cues remains limited, although it is now well appreciated that material cues modulate cell functions. Here, an overview of how the regulation of different aspect of cell metabolism, including energy homeostasis, oxygen homeostasis, and redox homeostasis could contribute to modulation of cell function is provided. Furthermore, recent evidence demonstrating how material cues, including the release of inherent metabolic factors (e.g., ions, regulatory metabolites, and oxygen), tuning of the biochemical cues (e.g., inherent antioxidant properties, cell adhesivity, and chemical composition of nanomaterials), and changing in biophysical cues (topography and surface stiffness), may impact cell metabolism toward modulated cell behavior are discussed. Based on the resurgence of interest in cell metabolism and metabolic regulation, further development of biomaterials enabling metabolic regulation toward dictating cell function is poised to have substantial implications for regenerative engineering.

## 1 Introduction

At the leading edge of regenerative engineering, a convergence of stem cell science, developmental biology, and advanced materials design, to support clinical translation^1^ of biomaterials are playing a central role in revolutionizing this area of study in guiding the development of novel tissue repair strategies, medical devices, and drug delivery systems for the regeneration of complex tissues. The growing demand of biomaterials in regenerative medicine calls for increased investigation to develop a comprehensive understanding of the fundamental mechanisms underlying cell responses to biomaterials. Studies using materials designed to recapitulate individual aspects of the cell–material interface, a complex and dynamic microenvironment,^2^ repeatedly illustrate a variety of altered intracellular events shifting cell behavior as a result of the cells' capability to sense and integrate material cues.^2^, ^3^, ^4^ However, a full picture of the relationship between a cell and its surroundings is far from complete, as exemplified by limited understanding of how the intracellular metabolic pathways are influenced by material‐derived cues, especially when cell metabolism is no longer considered as a bystander but as a series of intracellular events of cells that dynamically crosstalk with signaling and gene expression to influence their decision‐making.^5^, ^6^, ^7^, ^8^ Indeed, recent studies have advanced the hypothesis that the intrinsic properties of synthetic materials may influence cell metabolism potentially directing cell behavior to impact regenerative engineering outcomes by means of releasing soluble metabolic regulatory factors (e.g., ions, degradation products, and oxygen), incorporating antioxidative properties, and tuning cell adhesion, chemical composition, topography and material stiffness.

In this review, we intend to offer an overview of 1) the comprehensive and emerging understanding of metabolic regulation and how it may crosstalk with signaling and gene expression to dictate cell behavior; 2) how key aspects of the metabolic state of the cell (i.e., energy homeostasis, oxygen homeostasis, and redox homeostasis) could be regulated, particularly focusing on the regulatory role of metabolite and its implications in regenerative engineering; and more importantly, 3) recent evidence supporting the notion that materials properties may be engineered to regulate cell metabolism, and how these findings can possibly be exploited in aims to inspire innovation for the next generation of biomaterials that dynamically communicate with intracellular metabolic activities toward deliberated and improved regenerative outcomes.

## 2 Metabolic Regulation in Regenerative Engineering

### 2.1 Cell Metabolism and Metabolic Regulation

**Figure 1:**
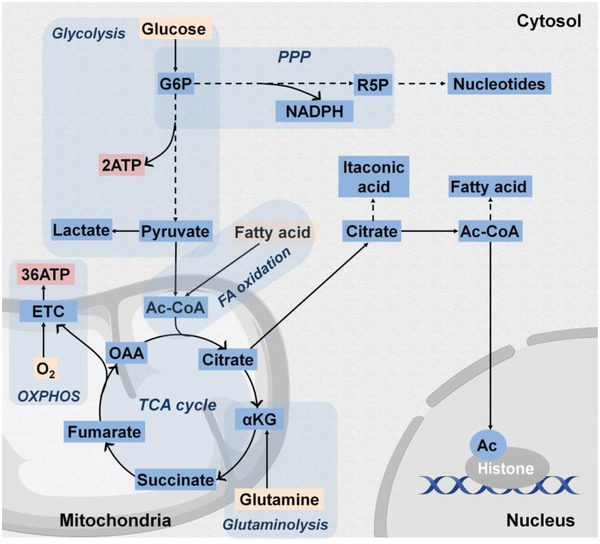
Overview of the core cell metabolic pathways. The energy source, ATP, is imperative for cell survival, proliferation, differentiation, and cell‐specific functions. ATP generation is derived from the intracellular processes, glycolysis, and cellular respiration. Glucose, a primary energy substrate, is imported into the cytosol, from the extracellular space and undergoes conversion into pyruvate via a series of chemical reactions collectively known as glycolysis obtaining a net of 2 ATP per mole of glucose. Pyruvate is converted into acetyl–CoA (Ac–CoA) in mitochondrial to enter the tricarboxylic acid (TCA) cycle, which drives the electron transfer chain (ETC) yielding a net production of 36 ATP molecules in a process called oxidative phosphorylation (OXPHOS). Notably, in certain cell types, such as cancer and endothelial cells, glycolysis‐derived pyruvate molecules are converted into lactate even when ample O_2_ is available, a phenomenon referred to as aerobic glycolysis. The glycolytic flux can also be directed through the pentose phosphate pathway (PPP) to generate NADPH, a cofactor for redox homeostasis and cellular respiration, as well as ribose‐5‐phosphate, a substrate for nucleotide synthesis. Furthermore, alternative macromolecules can feed into the TCA cycle besides pyruvate exhibited by conversion of fatty acids to acetyl–CoA via β‐oxidation and by conversion of glutamine to α‐ketoglutarate (α‐KG) via glutaminolysis. TCA cycle citrate may also be exported to the cytosol where it serves as a substrate for itaconic acid synthesis in M1 macrophages or may be converted to acetyl–CoA for fatty acid synthesis. Nucleocytoplasmic acetyl–CoA is additionally required as a substrate for histone acetylation (Ac) of chromatin histones, which impacts chromatin structure and gene transcription.

Cell metabolism is a compilation of enzyme‐catalyzed chemical reactions occurring within cells essential to all living organisms. It involves the breakdown of nutrients to generate energy in the form of adenosine triphosphate (ATP) (catabolism) as well as the consumption of energy to synthesize complex molecules needed to execute cellular activity and for energy storage (anabolism). Glucose is the primary substrate used to fuel cellular respiration in glycolysis and oxidative phosphorylation (OXPHOS). Glycolysis involves the conversion of glucose to pyruvate in the cytoplasm with a net production of two ATP molecules per mole of glucose. The entry of pyruvate into the mitochondrial matrix manifests the transition from glycolysis to the tricarboxylic acid (TCA) cycle (**Figure** **1**) generating electron carriers, such as nicotinamide adenine dinucleotide (NADH) and flavin adenine dinucleotide (FADH_2_), which donate electrons to the mitochondrial electron transport chain (mETC) located at mitochondrial inner membrane during oxidative phosphorylation (OXPHOS), oxygen (O_2_) is the final electron acceptor in the mETC producing water and is critical to the OXPHOS process. A net amount of 36 ATP molecules are produced by OXPHOS. Cells also have the flexibility to metabolize other substrates besides glucose when available in the local microenvironment, such as fatty acids,^9^ or glutamine^10^ to replenish the TCA cycle. To maintain metabolic homeostasis, cells have evolved tightly regulated mechanisms to modulate metabolic flux.^7^, ^8^, ^11^ In response to hormones and other extracellular factors (e.g., growth factors) that communicate signals between tissues, cells adjust metabolic activity and pathways via affecting the expression of transporters and metabolic enzymes through modulating gene expression, mRNA transcription and translation, allowing for context‐specific metabolic adaptation to support physiological functions induced by the cell signaling.^6^, ^7^ For example, when exposed to osteogenic signals, osteoblast progenitor cells immediately increase glucose uptake by elevating the expression of glucose transporter 1 (Glut1), a glucose transporter, to meet the energetic needs of osteogenic differentiation.^12^, ^13^ Moreover, increased consumption of glutamine together with elevated expression of glutamine catabolic enzymes have also been reported.^10^ The availability of metabolic cofactors, a class of nonprotein chemical compounds (e.g., acetyl–CoA introduced in Section 2.2, and citrate introduced in Section 3.1) or metallic ions (e.g., Fe^2+^ and Co^2+^ introduced in Sections 2.3 and 2.4, respectively) required for enzyme function, could also directly or indirectly affect enzyme activity.

Metabolic flux, in turn, strikes back to influence state of the cell^6^, ^14^ with increasing evidence that changes in the metabolic state can instruct both signaling pathways and gene expressions.^5^, ^6^, ^15^, ^16^ Particularly, how metabolites directly impact a myriad of processes from signaling to gene expression in cells has been increasingly appreciated^6^, ^15^ and will be focused on in the present review. A detailed review of the possible role of metabolic enzymes in regulating metabolism, transcription, and epigenetics has been reported previously by van der Knaap and Verrijzer.^6^ Specifically, posttranslational modification (PTM) of proteins using metabolites as substrates provides a mechanism for cells to sense metabolite levels to facilitate the influence of specific metabolic pathways on signal transduction pathways.^14^ For example, glycolytic flux, glutamine consumption, and acetyl–CoA availability are all required for N‐linked glycosylation as well as folding and function of growth factor receptors, which provides a mechanism to couple metabolite availability with growth factor‐mediated signaling.^17^ In addition, metabolites themselves may function as signaling molecules, exemplified by the well‐known ATP signaling.^18^, ^19^, ^20^ Other intermediate metabolites, such as succinate, also have been realized to exhibit extrametabolic function enhancing the immunity of dendritic cells by triggering intracellular calcium release and inhibiting cyclic adenosine monophosphate (cAMP)^21^ upon binding to the GPR91,^22^ a G protein–coupled receptor on the dendritic cells. PTMs of chromatin histone proteins, such as histone acetylation using glucose‐derived acetyl–CoA (introduced in Section 2.2) as the substrate^23^ alters the chromatin structure to modify its binding capabilities with particular transcription factors, suggesting an intimate link between extracellular nutritional changes, intracellular metabolic flux, and gene expression.^24^ The above novel insights challenge the long‐held assumptions that all metabolic fluxes provide identical housekeeping functions ubiquitous to all cells, provoking a revived interest in cell metabolism and metabolic regulation.

While the basic metabolic pathways remain the same, the regulation of metabolism has to be considered in context of the relevant tissue‐specific metabolic milieu^5^ as nutrient availability (glucose, amino acids, and lipids), oxygen availability, metabolite availability, and radical oxygen species (ROS) level in the microenvironment vary between different cells. This variability in local cell environment is particularly evident in regenerative engineering scenarios as tissue damage often destroys the vascularization network necessary for efficient nutrient and oxygen delivery and results in an accumulation of ROS. Similarly, cells are able to actively remodel their microenvironment via metabolic pathways by exporting intermediate metabolites into the surrounding extracellular space either as signaling molecules (e.g., extracellular ATP and its derivative nucleosides known to get released by cells and bind purinergic receptors to activate intricate signaling pathways^18^, ^19^, ^25^) or to directly participate in the cells' physiological function (e.g., TCA cycle citrate is exported extracellularly to regulate osteoblast mineralization and deposit in bone minerals as an integral part of apatite nanocomposite^26^). Accordingly, in the following sections, the metabolic relationship between a cell and its surroundings is elucidated giving particular attention to how fundamental aspects of the cell metabolic state, like energy homeostasis, oxygen homeostasis, and redox homeostasis may be modulated in response to environmental factors.

### 2.2 Metabolic Regulation of Cell Energy Homeostasis

Cell energy homeostasis is a cellular process balancing energy production with energy consumption predominantly through nutrient uptake and biosynthesis, respectively. Glycolysis and OXPHOS are the two major metabolic processes converting nutrients to energy in the form of ATP for cells to support biosynthetic activities. Reprogramming of the energy metabolism in stem cells during self‐renewal and differentiation (see reviews^8^, ^27^, ^28^), endothelial cells during angiogenesis (see review^29^, ^30^, ^31^), and immune cells during activation (see review^32^, ^33^) have received tremendous attention in recent years. A summary of the characteristic metabolic profiles of various cell types and lineages is provided especially those involved in key events largely affecting regenerative engineering outcomes, such as in stem cell differentiation, angiogenesis, and immune response (**Table** **1**). For example, undifferentiated mesenchymal stem cells (MSCs) residing in hypoxic environments in vivo exhibit heightened glycolysis activity and lowered OXPHOS activity,^34^ which is shown to prevent senescence resulted from oxidative stress and thereby preserve MSCs for long‐term self‐renewal.^35^ Moreover, a metabolic shift from glycolysis toward elevated OXPHOS was found to be required for osteogenic differentiation and especially for the adipogenic differentiation process. In contrast, reduced O_2_ consumption and OXPHOS activity during chondrogenic differentiation indicated a shift toward increased glycolysis.^35^ More importantly, a continuous and uninterrupted hypoxic culture condition (2% O_2_) during MSCs differentiation, was found to reduce the extent of osteogenesis shown as limited ALP production and no calcium deposit, while with chondrogenic differentiation remained unaffected,^36^ suggesting the potential of directing MSCs differentiation through modulation of cell energy metabolism.^8^ Immune cells remain quiescent followed by rapid growth and proliferation upon activation.^33^ To accomplish this, lymphocytes, for example, in response to growth factor stimulation shift from a low metabolic state sustaining basal functions to a state of elevated glucose uptake and activated citrate synthase, which facilitates the production of citrate for fatty acid synthesis to support their rapid growth.^37^ On the other hand, differentiated cells, such as cardiomyocytes^9^ and neurons,^38^ have no potential of proliferation, but have a high demand for ATP and oxygen to maintain their physiological function, thereby relying heavily on OXPHOS to efficiently generate ATP to remain energy homeostasis.

**Table 1:** Summary of key active metabolic pathways for different cell types pertinent to regenerative engineering outcomes

Cell type	Cell name	Metabolic phenotype	Ref.
Stem cells	Induced pluripotent stem cells (iPSCs)	Anabolic glycolysis, PPP	8
	Mesenchymal stem cells (MSCs)	Low glycolysis	8, 27, 35
Endothelial cells	Tip cells	Increased aerobic glycolytic (stimulated by VEGF)	29, 30
	Stalk cells	Aerobic glycolytic; fatty acid catabolism for nucleotide biosynthesis	29, 30
	Phalanx cells	Low aerobic glycolysis	29, 30
Immune cells	Neutrophils	Aerobic glycolysis; PPP	32, 33
	M1 macrophage	Aerobic glycolysis; PPP	32, 33, 73
	M2 macrophage	Fatty acid oxidation	32, 33, 73
	Activated dendritic cells (DCs)	Aerobic glycolysis; PPP	32, 33
	Resting T cells	Low glycolysis; low OXPHOS	32, 33
	Activated T cells	Aerobic glycolysis	32, 33
	Regulatory T cells (T_reg_)	Fatty acid oxidation	32, 33
	Memory T cells	Fatty acid oxidation	32, 33
Differentiated cells	Osteoblasts	High OXPHOS; glutaminolysis	28, 34
	Adipocytes	High OXPHOS; high ROS	28, 34
	Chondrocytes	High glycolysis	28
	Neurons	High OXPHOS	38
	Cardiomyocytes	High OXPHOS; fatty acid oxidation	9
	Myoblasts	Anabolic glycolysis; PPP	28

Abbreviations: PPP, pentose phosphate pathway; VEGF, vascular endothelial grow factor; OXPHOS, oxidative phosphorylation; ROS, reactive oxygen species.

Limited glucose, the major energy substrate for all cells, has been linked to reduced cell energy levels and impaired cellular functions like suppressed bone formation,^12^ inactive sprouting of endothelial cells,^39^, ^40^ and poor survival of lymphocytes.^41^ A common way for cells to acutely adapt to low glucose levels is to increase the oxidation of alternative energy substrates available in the microenvironment, such as amino acids (e.g., glutamine^10^), fatty acids,^9^ and metabolites (e.g., lactate,^42^ pyruvate,^39^ and citrate^43^) to meet their energy needs. In terms of signal transduction, AMP‐activated protein kinase (AMPK) serving as an energy sensor^12^ and mammalian target of rapamycin (mTOR) as an amino acid sensor also have been described to coordinate energy metabolism with cell function.^27^ Specifically, AMPK, in response to changes in ATP/AMP and ATP/ADP ratios, coordinates diverse metabolic responses to balance energy production and consumption.^44^ It is stimulated by energetic stress (e.g., a low ATP/AMP ratio) to activate ATP‐producing catabolic pathways while repressing anabolic reactions through controlling the activity of numerous proteins via phosphorylation for short‐term regulation and by regulation of gene expression for long‐term regulation.^5^ An example of this is observed when glucose uptake is compromised during osteoblast differentiation; a decrease in ATP production activates AMPK, which subsequently promotes the degradation of Runx2, the predominant osteogenic transcription factor, thereby inhibiting highly anabolic osteogenesis.^12^

In addition to ATP production, cell energy metabolism provides intermediate metabolites as key substrates including carbon sources (e.g., acetyl–CoA),^45^ nitrogen sources (e.g., glutamine)^39^ and reducing agents (e.g., NADPH) for biosynthesis,^46^ as well as cofactors for chromatin structure modification.^47^ Acetyl–CoA represents a pivotal metabolite (Figure 1), and its cellular level reflects the metabolic state of cells in response to a number of microenvironment factors, such as nutrient availability and oxygen availability.^6^, ^48^ Acetyl–CoA is generated from mitochondrial‐derived citrate and serves as a direct precursor in the cytosol for fatty acid synthesis. More importantly, nucleocytoplasmic acetyl–CoA is also used as substrate during the acetylation of chromatin histones to regulate chromatin structure and gene transcription.^23^ In the absence of glucose or oxygen, total histone acetylation levels are substantially reduced.^49^, ^50^ Uptake of extracellular acetate may compensate for this deficiency to maintain global histone acetylation for rescued lipid synthesis and adipocytes proliferation,^50^ improved cancer cell survival under hypoxic conditions,^45^ and promoted glioblastoma cell adhesion and migration.^47^ Together, the above studies demonstrate the intriguing concept that the regulation of cell energy metabolism could be the determining factor of cell survival, proliferation, differentiation, and specific functions as a cell undergoes a shift in phenotype. There is also a critical need to rigorously characterize the metabolic environment of each cell type to better understand their specific energy and biosynthetic needs providing guidance for the design of regenerative engineering strategies.

### 2.3 Metabolic Regulation of Cell Oxygen Homeostasis

The maintenance of oxygen homeostasis is essential for the survival and function of most cells since oxygen is needed in the OXPHOS process for ATP production and is required for antimicrobial effector enzymes (e.g., nitric oxide synthase) for O_2_‐dependent antimicrobial defense.^51^ Oxygen shortage occurs when the distance between cells and blood vessels exceeds 100–200 µm.^52^ Tissue damage that destroys the vascularization network for efficient oxygen delivery is a major cause of tissue hypoxia. Acute hypoxic environments could lead to a metabolic shift to anaerobic metabolism and energy conservation. Meanwhile, sustained hypoxic conditions often result in extensive cell death and tissue necrosis. More importantly, various cells with high metabolic activity and oxygen demands, such as cardiomyocytes^9^ and neurons^38^ (Table 1), are remarkably sensitive to hypoxic conditions. Therefore, it is imperative to deliver adequate oxygen supply to the hypoxic tissue and/or regulate cell metabolism to accommodate the hypoxic environment.^53^

**Figure 2:**
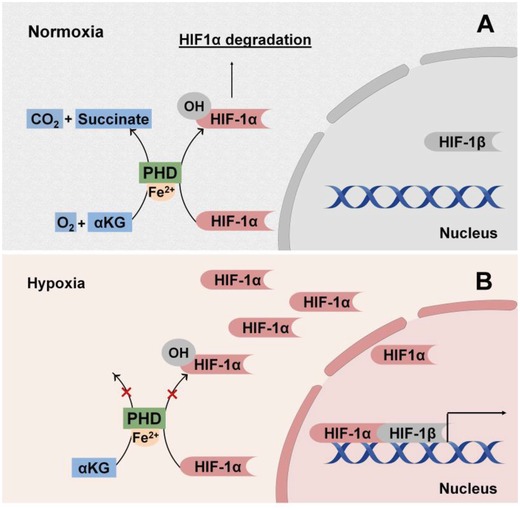
Oxygen‐dependent regulation of hypoxia‐induced factor (HIF) signaling. A) In normoxic environment, prolyl hydroxylase domain enzymes (PHDs) hydroxylate HIF‐1α subunits with production of carbon dioxide (CO_2_) and succinate as byproducts. Hydroxylation requires α‐KG and oxygen (O_2_) as substrates and is catalyzed by ferrous ions (Fe^2+^). The hydroxylated HIF‐1α subunits then undergo ubiquitination and degradation preventing nuclear translocation. B) In hypoxic conditions, there is an insufficient O_2_ supply for extensive hydroxylation of HIF‐1α subunits avoiding degradation to translocate to the nucleus and complex with HIF‐1β to promote transcription.

Hypoxia‐inducible transcription factor (HIF) is one of the most well‐known transcription factors that mediates oxygen‐sensitive signaling pathway and can be further modulated by metabolic regulation. HIF behaves as the transcriptional oxygen sensor that is precisely mediated by oxygen‐dependent posttranslational regulation of HIF‐α subunits by a family of prolyl hydroxylase domain containing enzymes (PHDs). When O_2_ levels are high, HIF‐α is hydroxylated by PHDs, and then the hydroxylated HIF‐α undergoes ubiquitylation and subsequent degradation by proteasome.^54^ In hypoxic conditions, O_2_ becomes limited for PHDs activity, and the HIF transcription factor is stabilized and accumulates to trigger gene expressions (**Figure** **2**). In addition to O_2,_α‐ketoglutarate (α‐KG) and Fe^2+^ are also required by PHDs for the hydroxylation of HIF‐α. Accumulation of succinate and fumarate, both of which are TCA cycle intermediate metabolites,^55^ have been found to stabilize HIF‐α by inhibiting PHDs. In fact, other metabolites, such as citrate and oxaloacetate in TCA cycle (Figure 1),^56^ have also been linked to HIF‐α stabilization by inhibiting PHD activity suggesting a potential intimate link between mitochondrial metabolism and HIF signaling. In addition, Fe^2+^ is required for PHD activity, which connects oxidative stress with HIF stabilization because elevated ROS levels could oxidize intracellular Fe^2+^, resulting in activated HIFα‐dependent transcription.^57^

Upon HIF stabilization, HIF mediates adaptive responses to hypoxia, including angiogenesis,^58^ metabolic reprogramming, and O_2_‐independent host defense.^59^ Angiogenesis represents a local tissue response to decreased oxygen levels. Upon stabilization, HIF is translocated to nucleus and then orchestrates the transcription of multiple genes encoding angiogenetic growth factors and cytokines, such as angiopoietin 1 (ANGPT1),^60^ vascular endothelial growth factor (VEGF),^61^ and platelet‐derived growth factor B (PDGFB).^62^ Indeed, TCA cycle metabolites, such as citrate,^63^ succinate,^64^ and oxaloacetate^65^ have demonstrated the potential of inducing angiogenesis by elevating VEGF expression, although whether HIF stabilization is the underlying mechanism remains to be investigated. Of note, HIF signaling also crosstalks with nutrient and redox metabolism to preserve energy and redox balance. HIF‐1α stabilization by pharmacological inhibition or knockout of PHDs promotes glutamine‐derived glutathione production to maintain redox homeostasis during oxidative stress while enabling glycogen‐dependent bioenergetics during glucose deprivation, which together resulted in improved postimplantation bone cell survival and substantially enhanced bone regeneration,^66^ highlighting the clinical translational implication of metabolic regulation of cell oxygen homeostasis in regenerative engineering.

### 2.4 Metabolic Regulation of Cell Redox Homeostasis

A balanced redox state of cells and the surrounding microenvironment have been extensively linked to tissue physiological function and regenerative engineering outcomes.^67^ ROS levels can rise as a consequence of changes in nutrient and oxygen availability or other stresses eliciting cell damage.^68^ The implantation of biomaterials could also trigger an inflammatory response to some extent, resulting in the production of ROS.^69^ Prolonged excessive levels of ROS could be deleterious causing significant destruction of cellular structures and increased cell death possibly leading to a burst release of ROS to the microenvironment affecting surrounding cells, which in many cases, results in necrosis, inflammation, and fibrosis.^70^ Although, excessive ROS have been linked to exacerbating pathological processes as well as implant and cellular transplant failure, evidence also suggests a favorable role of ROS for regulating vital cellular processes, such as stem cell renewal and differentiation.^71^ For example, endogenous ROS is necessary to initiate adipogenesis, suggesting that OXPHOS and ROS are causal factors in promoting the differentiation process.^72^ Moreover, ROS produced from immune cells (e.g., neutrophils and M1 macrophages) exhibiting antimicrobial effects represent ROS as a well‐known component in the host defense toward microbial invasion.^73^ Therefore, cellular redox state has to be intricately regulated to maintain healthy cellular functions while preventing oxidative damage.

**Figure 3:**
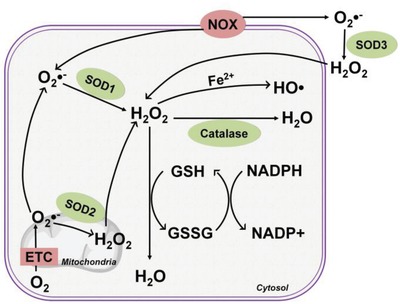
Regulation of redox homeostasis. Primary intracellular production of radical oxygen species (ROS) is derived from the metabolic mitochondrial ETC and membrane‐bound NADPH oxidase (NOX). Enzymes, such as superoxide dimutases (SODs) and catalase, in conjunction with antioxidant molecules, reduced glutathione (GSH), and NADPH, perform critical roles in the endogenous antioxidant defense system to preserve redox homeostasis. Anion superoxide (O_2_•^−^) is the leading form of produced ROS, which is rapidly converted into cell permeable hydrogen peroxide (H_2_O_2_) by SOD2 in the mitochondria, by SOD1 in the cytosol, and extracellularly by SOD3. H_2_O_2_ can be catalyzed to the most reactive hydroxyl radicals (HO•) in the presence of Fe^2+^ (Fenton reaction) or be converted into water (H_2_O) by catalase. The reduced form of glutathione (GSH) and the oxidized form of glutathione (GSSG), together with a reducing agent (e.g., NADPH) represent another major antioxidant mechanism converting radical H_2_O_2_ to H_2_O.

Of note, cell metabolism possesses an intimate connection and reciprocal crosstalk with cellular redox homeostasis. Cell respiration represents a major source of ROS as O_2_ is used as the ultimate electron acceptor during OXPHOS increasing the risk of generating ROS intermediates in the mETC (**Figure** **3**) like the superoxide anion (O_2_•^−^) and hydrogen peroxide (H_2_O_2_). H_2_O_2_ can also be converted into the hydroxyl radical (HO•), in the presence of Fe^2+^, via a process known as Fenton's reaction.^74^ Moreover, NAPDH oxidases (NOX) produce ROS in the form of O_2_•^−^ in the cytoplasm as well as in the cell milieu. Cells have evolved endogenous antioxidant defense mechanism to minimize ROS generation and support antioxidant scavenging.^75^ The most relevant antioxidant enzymes include superoxide dismutases (SODs) that convert superoxides to less reactive H_2_O_2_, and catalase, which reduces H_2_O_2_ into water and oxygen.^75^ Redirection of core metabolism into specific metabolic routes (e.g., pentose phosphate pathway (PPP) and the glutaminolysis) is also involved in the antioxidant defensive mechanism.^5^ For example, reduced glutathione (GSH), one of the key endogenous antioxidant molecules, is synthesized using glutamine‐derived glutamate as the precursor (Figure 1). Moreover, glutamine deprivation has been found to impair cell proliferation in part by elevating intracellular ROS levels in endothelial cells accompanied with reduced total GSH levels, pointing out a direct link between glutamine metabolism and redox homeostasis in vessel sprouting.^29^ Interestingly, the balance of ROS generation and removal is also dynamically modulated during cell differentiation.^72^ For example, directed adipogenesis of human mesenchymal stem cells (hMSCs) increases mTORC1‐dependent mitochondrial biogenesis leading to increased OXPHOS accompanied with elevated ROS production, which is required to promote the adipo‐differentiation process.^72^ On the other hand, osteoblasts, which are less tolerant to ROS than that of adipocytes, also upregulate OXPHOS during differentiation, together with simultaneously increased expression of antioxidant enzymes, such as catalase and SODs,^34^ suggesting the distinct redox metabolic phenotype of osteoblasts compared to that of adipocytes.

## 3 Harnessing Biomaterial Cues for Metabolic Regulation

**Figure 4:**
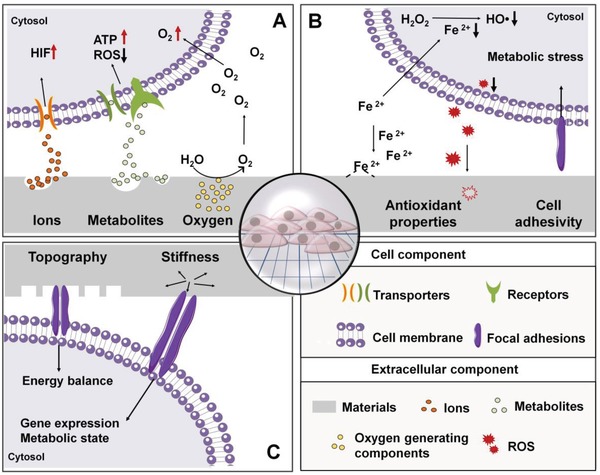
Overview of biomaterial‐based regulation of cell metabolism. Metabolic regulation from biomaterials design may be achieved by A) releasing metabolic regulators (e.g., metal ions, metabolites, and oxygen), which subsequently enter cells to modulate intracellular metabolic activities; by B) introducing antioxidative properties (e.g., ion chelation or ROS “quenching”) to or altering cell adhesivity of the materials through chemical modification to modulate external factors indirectly impacting intracellular redox homeostasis; and by C) modulating the biophysical properties of the base materials via the design of surface features and stiffness alteration to provide biophysical cues which are converted to biochemical cues involved in metabolic regulation.

The intriguing concept of cell metabolism crosstalk with signaling and gene expression to modulate cell function may be leveraged in advancing of biomaterials research and regenerative engineering fields, especially considering biomaterials designed to recapitulate the cell microenvironment have repeatedly provided strong evidence that biomaterial cues can dynamically affect cell behavior.^2^, ^3^ Therefore, it is speculated that strategies of modulating metabolic status may be achieved through deliberate materials design to induce material‐derived cellular cues as summarized in Table 3 and illustrated in **Figure** **4**, via 1) the release of inherent metabolic regulators from biomaterials; 2) tuning the chemical properties of the base biomaterials; 3) modifying the physical properties at the cell–material interface.

### 3.1 Release of Inherent Metabolic Regulators

Intuitively, one can envisage that the releasing of metabolic regulators, such as ions, metabolites and O_2_, which are inherently embedded or incorporated in biomaterials, may modulate intracellular metabolic flux after entering cells. In fact, metabolic regulator release represents the primary conduit through which biomaterials affect cell metabolism^76^, ^77^, ^78^, ^79^, ^80^ and subsequently influence cell functions, which is of particular interest for regenerative engineering.

#### 3.1.1 Release of Metal Ions

Metal ions serve as common cofactors for metabolic enzyme activity to modulate enzyme activities. Thus, ion‐doped biomaterials enabling controlled release of ions, such as Ca^2+^, Mg^2+^, Zn^2+^, Co^2+^, and Cu^2+^, which directly serve as enzyme cofactors or indirectly affect enzyme activity via substitution of key ion cofactors, may possess great potential in regulating metabolism toward modulated cell function in regenerative engineering. One of the most noted examples is a set of metal ions, such as Co^2+^, Cd^2+^, Cu^2+^, and Mn^2+^, that competitively replace the iron located at the active center of iron‐dependent enzyme PHDs, which thereby leads to the stabilization and activation of HIF‐1α as introduced in Section 2.3. Among all the ions tested, Cu^2+^ and Mn^2+^ stabilize HIF only at high concentrations (**Table** **2**), while Co^2+^ and Cd^2+^ showed the highest amplitudes of HIF activation (5–7‐fold over control). Given Cd^2+^ is a toxic heavy metal ion,^81^ Co^2+^ is a more favored ion for HIF‐1α stabilization by inhibiting HIF‐1α degradation, Therefore, Co^2+^‐doped bioactive glass was designed to release Co^2+^ in a controlled manner, which enhanced HIF‐1α activity in a concentration‐dependent manner, as expected, leading to improved hMSCs survival and elevated VEGF expression in hMSCs.^76^ Resorbable Co^2+^‐doped bioactive glass was processed into microparticles (38–100 µm) and embedded into collagen–glycosaminoglycan scaffolds for bone repair applications. The addition of the Co^2+^‐doped bioactive glass particles greatly enhanced the VEGF gene expression and production from endothelial cells in a dose‐dependent manner in comparison to its counterpart without Co^2+^. Moreover, the Co^2+^‐treated group supported osteoblast proliferation and osteogenesis, displayed as increased alkaline phosphatase (ALP) production at day 7 and substantially enhanced calcium deposition at day 28.^77^ Similarly, Cu^2+^ has also been doped into biomaterials, such as in bioactive glass,^82^ bioactive silicate (13‐93) glass,^78^ and a graphene‐based composite,^79^ to activate HIF‐1α with a resulting increase in secretion of VEGF and BMP‐2 for promoted angiogenesis and osteogenesis.

**Table 2:** Summary of the effective and toxic concentrations of the released biomaterials cues

Released cues	Effective concentrations	Toxic concentrations
Metal ions		
Co^2+^	25 × 10^−6^ m for HIF stabiliztion in neuroblastoma cells;^119^ 3–12 ppm for HIF activation in endothelial cells^77^	1–2 × 10^−3^ m (acute toxic concentrtaion to lung cells^120^)
Cd^2+^	7 × 10^−6^ m for HIF stabiliztion in neuroblastoma cells^119^	15 × 10^−6^ m (LD50 for pituitary cells^81^)
Cu^2+^	100–200 × 10^−6^ m for HIF stabiliztion in hepatoma cells^121^	≈200 × 10^−6^ m (IC50 for hepatoma cells^121^)
Regulatory metabolite		
Citrate	100–2000 × 10^−6^ m for promoted osteo‐phenotype progression of hMSCs^80^	37.3 × 10^−3^ m (EC50 for hMSCs^86^); 10.9 × 10^−3^ m (EC50 for MG63^86^); 10.5 × 10^−3^ m (EC50 for 3T3^86^);
Inorganic phosphate	5 × 10^−3^ m for induced osteogenic differentiation of hMSCs^19^	Not available
Lactate	0.06–0.17 mg mL^−1^ for radical scavenging^92^	20 × 10^−3^ m (critical toxic concentration for hMSCs^122^)

#### 3.1.2 Release of Regulatory Metabolite

**Figure 5:**
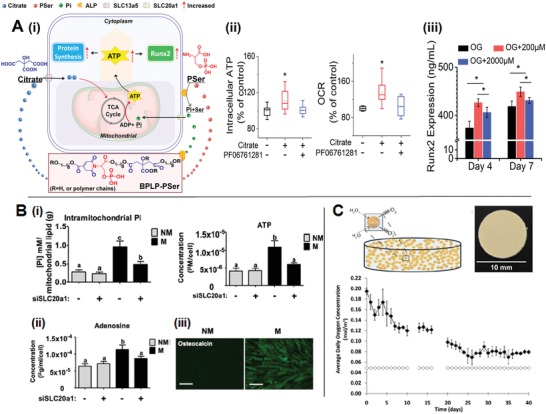
Metabolic regulator release from biomaterials. A) i) Schematic illustration of the citrate‐mediated metabonegenic mechanism in a human mesenchymal stem cell (hMSC) resulting from citrate release upon citrate‐based biomaterial degradation, ii) elevated intracellular ATP levels via modulation of the major energy‐producing pathways (e.g., glycolysis and OXPHOS) by citrate, which subsequently iii) promoted the Runx2 mediated osteo‐differentiation.^80^ B) Inorganic phosphate (Pi) released from resorbable calcium phosphate was found to i) enter cells to reach the mitochondria where it serves as the direct substrate for ATP syntheses. The cumulated ATP is secreted from cells and ii) degraded to adenosine, which in turn impacts osteogenesis as exhibited by iii) osteocalcin production via autocrine/paracrine signaling (scale bar: 100 µm) (Adapted with permission.^19^ Copyright 2013, PNAS). C) Oxygen‐generating materials can be designed by embedding oxygen‐forming compounds, like CaO_2_, into hydrophobic polymers, such as PDMS. A PDMS disk containing 25% w/w CaO_2_ after soaking in buffer saline could lead to sufficient oxygen generation up to 6 weeks (Adapted with permission.^100^ Copyright 2012, PNAS).

A growing body of evidence has revealed that degradable biomaterials can deliver signals to cells via degradation products.^2^ Within the pool of degradation products that are gradually and dynamically released into the extracellular space in situ overtime, there may be metabolic regulators including regulatory metabolites, cofactors, and key substrates for energy production or biosynthesis that can impact intracellular metabolic events. Indeed, we recently identified citrate in the degradation products of citrate‐based biomaterials (CBBs) as this type of inherent metabolic regulator for hMSCs bioenergetics toward facilitated osteogenic differentiation via a mechanism named “metabonegenic” regulation (**Figure** **5**Ai).^80^ Intracellular citrate is well‐known as an intermediate metabolite, playing an important role in regulating energy homeostasis,^83^, ^84^ since it not only modulates the activity of key enzymes in both catabolic and anabolic pathways, but also could convert to acetyl–CoA, the direct substrate for fatty acid biosynthesis and histone acetylation.^15^, ^84^, ^85^ Meanwhile, citrate serves as a multifunctional and cytocompatible^86^ monomer, which contributes to the development of a family of versatile and functional CBBs with tunable mechanical and biodegradable properties.^87^, ^88^ It turned out that citrate released from CBBs during degradation entered hMSCs via plasma membrane transporter solute carrier familiar 13, member 5 (SLC13a5) to modulate two main energy producing pathways by elevating OXPHOS while inhibiting glycolysis, which ultimately resulted in significantly elevated intracellular ATP levels (Figure 5Aii).^80^ Given metabolic reprogramming from glycolysis to OXPHOS is required for osteo‐differentiation^35^ to meet the increasing energy demand, the citrate‐elevated cell energy status subsequently is favorable to fuel the high metabolic demand of hMSCs osteogenic differentiation, contributing to the accumulated Runx2 expression and an increase in production of bone‐related extracellular matrix (Figure 5Aiii).

In addition to regulatory metabolites, inorganic phosphate, serving as the direct substrate for ATP synthesis, was found in the degradation product of a mineralized matrix containing calcium phosphate prepared via biomineralization.^89^ More importantly, the extracellular inorganic phosphate could be uptake by hMSCs via a phosphate transporter SLC20a1, which was found to be directly involved in ATP synthesis resulting in an increase of intracellular ATP (Figure 5Bi).^19^ Instead of being used as an intracellular energy source, ATP was found to go exocytosis and degraded into adenosine (Figure 5Bii), an ATP metabolite, which subsequently promoted osteogenesis via the A2B adenosine receptors on cell surface by serving as an autocrine/paracrine signaling molecule (Figure 5Biii).^19^ Notably, inorganic phosphate can also be released from biomaterials with the addition of organic phosphates, like phosphoserine^80^ and glycerophosphate,^90^ under the action of cell‐derived alkaline phosphatase (ALP). It also explains the concerted effect of citrate and phosphoserine in their solute form as well as after incorporation into a new class of citrate‐based polymers, biodegradable photoluminescent polymers with phosphoserine (PSer) incorporated (BPLP‐PSer), in the elevation of intracellular ATP level, the enhancement of osteo‐differentiation progression in vitro and, more importantly, the accelerated bone repair in vivo.^80^ Consistent improvement in bone regeneration was also evident in poly(octamethylene citrate glycerophosphate) (POC‐GP), another type of citrate‐based materials by incorporating additional organic phosphates, particularly glycerophosphate–Ca, into citrate‐based materials,^90^ further supporting the notion that citrate and inorganic phosphate in the degradation product may play concerted role in regulating hMSCs energy metabolism toward facilitated bone regeneration.

Another way in which degradation products impact cell function is through modulating cell redox metabolism, such as by reducing the intracellular free radical species. For example, the lactate released from poly(ethylene glycol) (PEG) hydrogels that were copolymerized by PEG dimethacrylate and macromer poly(lactic acid)‐*b*‐PEG‐*b*‐poly(lactic acid) dimethacrylate during degradation has shown to be a free radical scavenger, which protected neuron progenitor cells from secondary damages that are induced by photoinitiator‐generated and cell‐produced free radicals.^91^ Although the underlying mechanism for the radical scavenging capability of lactate remains unknown, the effect is likely due to that soluble lactate, a byproduct of hydrogel breakdown, is able to be transported into cells as opposed to the lactate integrated into the initial hydrogel.^92^ Once in cells, lactate decreases the amount of intracellular ROS, leading to a more reduced intracellular redox state. An increase in reduced GSH content was also detected, which ultimately contributes to an marked improvement in cell survival and functions.^91^, ^92^ Similarly, long‐term release of radical scavengers including ascorbic acid,^93^ ferulic acid,^94^ and GSH,^95^ or antioxidant enzymes, like SOD (introduced in Section 2.4),^96^ incorporated into materials have also been shown to attenuate oxidative stress and improve cell survival upon release due to material degradation.

#### 3.1.3 Release of Oxygen to Maintain Oxygen Hemostasis

In addition to soluble ions and regulatory metabolites, biomaterials can be designed to generate and release O_2_ gradually over time (reviewed previously^53^) to cells in damaged tissues to relieve hypoxia‐induced tissue damage shown to prolong cell survival until endogenous neovascularization is achieved. The introduction of oxygen to biomaterials could be achieved mostly by incorporating oxygen generating components, which enables in situ oxygen generation. Sodium percarbonate (SPO) and peroxides (e.g., calcium peroxide (CaO_2_), magnesium peroxide (MgO_2_), and hydrogen peroxide (H_2_O_2_) are the most commonly used oxygen generating components.

For example, SPO is an adduct of sodium carbonate and hydrogen peroxide that, in the presence of water, spontaneously decomposes to generate oxygen. At biocompatible concentrations (1–2 mg mL^−1^), SPO has been found to efficiently sustain skeletal muscle metabolism under hypoxic conditions^97^ displayed by attenuated HIF accumulation, reduced glycogen depletion and unaltered contractility after 30 min of incubation under hypoxic conditions. An injection of SPO in a rat hindlimb ischemia model also preserved muscle metabolism and contractility, as loss of contractility is typically considered as a primary indicator of loss of metabolic homeostasis.^97^ Given that SPO is fast oxygen‐releasing component, in order to control the oxygen releasing rate, Harrison et al. incorporated SPO into PLGA films to prepare oxygen‐generating polymeric films,^98^ which were implanted around ischemic tissue in a mouse model for in situ production of oxygen. Indeed, the release of oxygen at a high rate was observed with the total oxygen generation almost completed after 24 h significantly decreased the hypoxic‐induced tissue necrosis and cell apoptosis for several days, as compared to the untreated control group.

The ultimate goal for the implantation of oxygen‐generating biomaterials is to deliver oxygen in a controlled and consistent manner to the damaged tissue until the neovascularization is achieved, which sometimes takes weeks.^53^ Also, the rate of oxygen release may impact the biocompatibility of the whole system.^53^ Therefore, a controlled and sustained release of oxygen for a suitable duration is highly desired, and there are a number of factors, such as pH, amount and type of oxygen generating components (i.e., particle size and solubility), and type of polymer (i.e., hydrophobicity and molecule weight), that have to be considered.^97^ For example, CaO_2_ solid particles have been incorporated into PLGA 3D scaffolds_,_ which prolonged oxygen release up to 10 days,^99^ and the increase of particle size could also lead to decreased initial burst release of oxygen.^53^ Encapsulation of oxygen generating compound into hydrophobic polymer has also shown to be an effective way to further slowdown the release of oxygen. For example, Pedraza et al.^100^ encapsulated solid CaO_2_, which produces oxygen when hydrolytically activated, within polydimethylsiloxane (PDMS), a highly hydrophobic and biocompatible polymer to fabricate implantable PDMS–CaO_2_ disks (Figure 5C). The utilizing of hydrophobic PDMS serving as a diffusional barrier to reduce the reactivity of CaO_2_, enabled a sustained oxygen generation from the PDMS–CaO_2_ disks for more than 6 weeks at an average rate of 0.026 × 10^−3^ m per day.^100^ The PDMS–CaO_2_ system greatly mitigated hypoxia‐induced cell death and preserved the metabolic function and the glucose‐dependent insulin secretion capability of β cells and pancreatic islet cells under hypoxic conditions, as comparable to that in normoxic controls. In addition to decreasing the burst oxygen release, further introduction of antioxidant enzymes such as catalase,^97^, ^99^ into the oxygen generating system has demonstrated its efficacy in removing possible reactive oxygen species by‐product during oxygen generation and thereby greatly improving the biocompatibility of the entire system.^99^ These results together strongly suggest the applicability and efficacy of sustained delivery of oxygen to maintain oxygen homeostasis in hypoxic scenarios commonly encountered in large damaged tissues, which is worthy of further validation in vivo to assess their potential for clinical translation.

### 3.2 Biochemical Cues of the Base Biomaterials

Several studies have demonstrated that chemical functionalization of the bulk biomaterial or of the material surface can direct cell response and behavior.^101^ First, the development of biomaterials functionalized with antioxidant moieties were motivated by antioxidant potential to interact with extracellular ROS and subsequently influence the intracellular redox state, which suggests a potential link between extracellular biochemical cues and intracellular metabolic activities. Recent studies about cell adhesivity^102^ through culturing cells on “stressful” surfaces (with poor adhesivity) resulted in metabolically stressed (accelerated mitochondria activity) cells further supporting the metabolic link with external materials stimuli. Finally, how to design the chemical composition of nanomaterials which have the potential to enter cells via endocytosis and impact intracellular metabolic reactions^103^, ^104^ is discussed in this section.

#### 3.2.1 Intrinsic Antioxidant Properties to Maintain Redox Homeostasis

**Figure 6:**
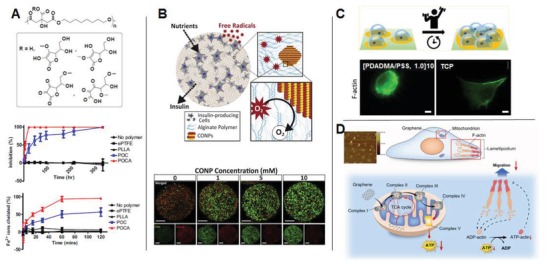
Biochemical cues from biomaterials for metabolic regulation. A) Citrate‐based biodegradable elastomers with inherent antioxidant properties poly(octamethylene citrate ascorbate) (POCA) (upper) was developed to possess (middle) strong radical scavenging activity and (lower) potent Fe^2+^ chelating capability (Adapted with permission.^93^ Copyright 2014, Elsevier). B) Cerium oxide nanoparticle (CONP)‐alginate composite hydrogel was developed for the encapsulation of β cells in which system CONP provided ubiquitous and renewable antioxidant protection from external ROS damage, resulting in greatly improved survival of β cells under superoxide exposure (scale bars: 200 µm) (Adapted with permission.^109^ Copyright 2011, RSC Publishing). C) Ultrathin polyelectrolyte multilayer ([PDADMA/PSS, 1.0]10) was designed to coat tissue culture plastic serving as a “biocompatible” but poorly adhesive substrate on which 3T3 fibroblasts exhibited a rounded morphology, diffuse organization of the actin cytoskeleton, stunted proliferation together with heightened metabolic stress (left) compared to that on tissue culture plastic alone (right) (scale bars: 10 µm). (Adapted with permission.^102^ Copyright 2018, ACS). D) Schematic illustration of how uptake of graphene nanosheets impairs the migration and invasion of metastatic breast cancer cells by disturbing electron transfer in the ETC and thereby reducing ATP production (Adapted with permission.^103^ Copyright 2014, Elsevier).

Aside from providing metabolic regulators to cells, biomaterials can also be designed to “take” cell‐generated or microenvironmental metabolic compounds by means of radical scavenging or ion chelation to thereby regulate the intracellular redox state of the cell. Given that metal ions participate in the formation of free radicals (e.g., Fe^2+^ serves as a catalyst for the Fenton reaction that produces hydroxyl radicals; see Section 2.4), the presence of specific metal ions may limit the beneficial effects of antioxidants, such as ascorbic acid. Therefore, the design of biomaterials that are capable of seizing the metal ions, especially Fe^2+^, from the microenvironment has been considered as one of the antioxidant strategies to locally attenuate oxidative stress. For example, citric acid with three carboxylic acid (–COOH) groups has shown potent ion‐chelating capabilities. Therefore, poly(octamethylene citrate) (POC)^93^ and water soluble poly(polyethylene glycol citrate‐*co*‐*N*‐isopropylacrylamide) (PPCN),^105^ belong to the citrate‐based biomaterials (CBBs) family, employ citric acid as the major antioxidative, multifunctional monomer^87^ and thereby are capable of chelating ions, scavenging free radicals, and inhibiting lipid peroxidation. Further incorporation of ascorbic acid into the POC network resulted in the development of a new polymer named poly(octamethylene citrate ascorbate) (POCA),^93^where the ascorbate incorporation synergized with citric acid by increasing the accessibility of the carboxyl and hydroxyl groups on citric acid leading to increased metal chelation onto POC (**Figure** **6**A). As expected, POCA also displayed almost complete abolishment of lipid peroxidation and further improved radical scavenging capacity compared to POC, probably resulted from the release of ascorbate during materials degradation suggesting its potential in regenerative engineering applications, especially where oxidative stress is a concern.

Another way for biomaterials to collect cell‐generated ROS to maintain redox homeostasis is derived from the capability of the material components to directly react with extracellular ROS^106^ or to mediate the conversion of noxious free radicals to a nontoxic counterpart.^107^ For example, cerium oxide nanoparticles (CONP) are known for their self‐renewing ability of redox cycle between Ce^3+^ and Ce^4+^ valence states, and for the potential to quench multiple types of free radicals.^108^ Addition of CONP at a concentration of 0.1 × 10^−3^ m into culture medium already potently protected β cells from oxidative damage. However, when the concentration reached 1 × 10^−3^ m, CONP could elicit marked cytotoxicity after being internalized by cells.^109^ To address the issue, Weaver et al. employed a simple strategy by engineering a nanocomposite hydrogel, which could highly retain CONPs within alginate hydrogel for extended period of time to minimize cell phagocytosis^109^ (Figure 6B). While using the composite system for β cell delivery, the presence of CONP in hydrogel provided protection to encapsulated cells without causing cytotoxicity, even when its concentration reached 10 × 10^−3^ m, which demonstrated its capability in preventing internalization of CONP by cells and in protecting the encapsulated β cells from free radical attack demonstrating its applicability in cell transplantation.^107^

#### 3.2.2 Cell Adhesivity

The adhesivity of cells to the surface of materials plays a critical role in modulating cell behavior and function. Most cells adhere to sense their substrate to preserve a normal morphology, cell homeostasis, and to proliferate. Surface chemical modifications, such as surface charging, hydrophilicity, and adhesion ligands, markedly determine cell adhesivity to the material surface. Interestingly, a recent study pointed out that cell adhesivity could also modify cell metabolism through affecting the energy balance. Specifically, thin films of alternately layered polyelectrolytes comprised of poly (4‐styrenesulfonic acid) (PSS) and poly (diallyldimethylammonium chloride) (PDADMAC) were designed, and by controlling the terminating or top layer, the [PDADMA/PSS, 1.0]_10_ surface was found to be biocompatible determined by live/dead assay but provided poor adhesivity for 3T3 fibroblasts. By culturing on such a stressful surface, the 3T3 cells encountered reduced focal adhesions, large macromolecular assemblies bridging material surface and cells,^102^ together with diffused organization of the actin cytoskeleton, as compared to that cultured on tissue culture plate (TCP) (Figure 6C) along with stunted proliferation on the polyelectrolyte film surface.^102^ It is worthy to note that a burst of metabolic stress that sustained for 5 days, defined as accelerated activity in mitochondria, was observed in cells cultured on the multilayer films with poor adhesivity.^102^ It seems that these cells must increase the production of certain components necessary for the development of cytoskeletal and focal adhesion complexes to stay weakly adhered on the multilayer surface.^102^ Given that generating additional cytoskeletal proteins and ECM consumes a lot of energy, it might provide explanation for the metabolic stress and reduced cell proliferation on poorly adhesive surfaces. Moreover, increased mitochondrial activity may be accompanied with the generation of ROS and inflammatory cytokines. Therefore, it is an important consideration to eschew from generating a stressful surface when designing synthetic surfaces for implants.

#### 3.2.3 Chemical Composition

To date, there is sparse evidence demonstrating that cells can sense chemical components of the base biomaterials in their solid form to modulate their intracellular metabolism, except studies in which the materials enter cells via endocytosis in forms of nanocarriers and become involved in metabolic chemical pathways suggest the possibility. For example, pristine graphene and graphene oxide (GO) nanosheets in dispersed form were designed to have an average length of 100–200 nm and a thickness of 3–4 nm, and after internalized by cells, they were found to suppress the activity of mETC complexes, which likely resulted in the disruption of electron transfer. It is probably due to the GO nanosheets have stronger capability to accept electrons than the mETC complexes.^103^ The disrupted mETC caused a significant decrease in ATP production, leading to impairment of F‐actin cytoskeleton assembly, the critical component for cancer migration and invasion, since high energy consumption is required for the biosynthesis of the cytoskeleton assembly. In view of this, PEG functionalized GO was developed to be applied as drug carrier, which after endocytosis was consistently found to inhibit the migration and invasion of human metastatic breast cancer cells^104^ through inhibiting the OXPHOS without altering glycolysis. It further was uncovered that the uptake of PEG–GO nanosheet by breast cancer cells not only disrupted the mETC,^103^ but also downregulated the key proteins involved in TCA cycle,^104^ which collectively impaired the migration and invasion of breast cancer cells (Figure. 6D).

### 3.3 Biophysical Cues of the Base Biomaterials

Cells can sense biophysical cues of local substrates, such as surface topography and stiffness, by forming focal adhesions and adjusting their cytoskeletal networks^110^ through guided cell behavior via signaling or epigenetic regulation.^3^ Although to date, few studies have investigated how extracellular biophysical cues can regulate metabolism toward altered behavior, available data suggest exciting possibilities, mainly through the conversion of biophysical signals into biochemical factors (e.g., ATP,^111^ ROS,^112^ and lineage‐specific metabolite^113^) that are involved in metabolic regulation.

#### 3.3.1 Surface Topography

It is well‐known that cells can sense and respond to surface topography of biomaterials, and that topographic cues, like geometry, roughness, and shape, in turn, profoundly influence cell morphology, migration, and differentiation. Although previous studies of topographic cues have not focused on identifying an impact on cell metabolism, evidence has implicated the potential link between extracellular topographic cues and their intracellular metabolic activities.^112^, ^114^, ^115^ For example, Singh et al.^114^ fabricated a PDMS surface containing micropatterned grooves with a spacing of 1 µm and a depth of 250 or 500 nm to study the response of astrocyte behavior. In addition to the aligned actin stress fibers and focal adhesions, astrocytes that were cultured on micropatterned surfaces showed enhanced mitochondrial activity accompanied by increased ATP release into the extracellular environment via lysosomal exocytosis, compared with that on flat surfaces. ATP, as a signaling molecule, further triggers elevated intracellular calcium intensity oscillation on microgroove patterns possibly suggesting enhanced astrocytes excitability with this regularly turbulent surface topography.^114^ However, the underlying reasons for topographical cues influencing cell metabolism and the relation to astrocyte function in central nervous system repair requires further investigation.

**Figure 7:**
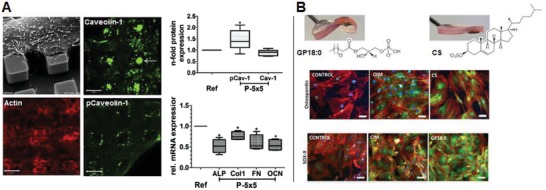
Biophysical cues from biomaterials for metabolic regulation. A) MG63 cells cultured on micropillars topography with a dimension of 5 µm × 5 µm × 5 µm and a spacing of 5 µm was found to drastically alter actin cytoskeleton organization and to induce attempted caveolae‐mediated phagocytosis of beneath micropillar evidenced by elevated caveolin‐1 expression and activation, which was accompanied with increased ROS and reduced ATP production leading to suppressed osteogenesis, as compared to that on flat surface (Adapted with permission.^115^ Copyright 2016, Elsevier). B) Supramolecular hydrogels of simple chemical functionality with tunable stiffness were designed to reveal stiffness‐related differentiation of pericytes/MSCs toward different lineages. In combination with metabolomics analysis, two types of lipid, the lysophosphatidic acid (GP18:0) in the glycerolipid pathway and the cholesterol sulfate (CS) in the steroid biosynthesis pathway, were identified and validated as key regulatory metabolites that may be involved in direct chondrogenic (shown as SOX‐9 expression) and osteogenic differentiation (shown as osteopontin expression), respectively (Adapted with permission.^116^ Copyright 2016, Elsevier).

In addition, certain surface features, including topographic protrusions and sharp edges, may also modulate cell metabolism to negatively impact cell function.^112^, ^115^ For instance, titanium surface was designed to display micropillar surface features (5 µm × 5 µm × 5 µm) with a spacing of 5 µm. When cultured MG63 osteosarcoma cells on such a surface, the attempt caveolae‐mediated phagocytosis of the underlying micropillars was observed in cells.^115^ Phagocytosis is an energy demanding process in various cell types, which means cells on the micropillar surface need to consume more ATP to support their attempt phagocytosis behavior, supported by the results showing a decrease in intracellular ATP level accompanied by an elevated ROS production (**Figure** **7**A). Impaired osteoblastic functions were also evident,^115^ displayed as a decrease in bone‐related matrix production, which are also energy consuming. Similarly, the surface topography is also considered as an indicator of the neutrophil death in response to the roughened surfaces of expanded polytetrafluoroethylene (ePTFE). On the roughened surface, there was a decreased cell viability with increased ROS production, although the mechanism underlying the phenomenon remains to be determined.^112^ Collectively, the above studies identify possible beneficial and adverse impacts that topographical cues may have on cell metabolism and behavior depending on the cell type and topography pattern, with a focus on the impact on cell energy and redox balance.

#### 3.3.2 Surface Stiffness

The concept that material mechanics including stiffness, elasticity and collagen density, can positively influence cell metabolism has emerged from cancer research and is receiving increased attention. It turns out that breast cancer cells (4T1 and 4T07) could reprogram their metabolism in response to the mechanics of their local extracellular matrix. Specifically, with an increase in collagen matrix density, the stiffness of the material increased with an exhibited decrease in oxidation of glucose and elevated oxidation of glutamine for the TCA cycle without affecting glycolysis,^116^ suggesting metabolic reprogramming occurred within cancer cells toward a more glycolytic signature. Consistent metabolic reprogramming of breast cancer cells, MDA‐MB231, was observed with increasing collagen substrate stiffness in another study.^117^ More importantly, when inhibiting myosin‐II contractility, the metabolism of cancer cells on stiff collagen substrate shifted back from glycolysis to OXPHOS, suggesting that mechanosensing network should be established for proper metabolic reprogramming.^117^ The intimate link between mechanosensing and metabolism may also provide explanations for how cells can sense the adhesivity of extracellular substrates resulting in metabolic stress as introduced in Section 3.2.2. Moreover, the sensitivity of metabolic reprogramming to substrate stiffness changing was also found to be cell‐type specific, as no metabolic change of nontumorigenic breast cells, MCF10A, was observed across all collagen substrates. The cell‐type specificity may be utilized for the development of novel therapy strategies to suppress cancer cell metabolism by targeting their mechanosensing sensitivity.

Substrate stiffness or elasticity of a material is known to bias the differentiation of MSCs cultured on it.^118^ Interestingly, a recent study further suggested the involvement of metabolic reprogramming, reflected by changes in nutrient utilization depending on the stiffness of hydrogel substrates. From the study using a self‐assembly nanofibrillar hydrogel with tunable stiffness, soft gels (1 kPa) induced multipotent pericytes to express neural marker while rigid gels (32 kPa) stimulated the expression of osteogenic markers, and growth on the stiff gel (13 kPa), resulted in expression of detectable chondrogenic markers.^113^ Furthermore, metabolomic analysis were employed for the first time to investigate the metabolic shifts on gels at 13 kPa and gels at 32 kPa up to 4 weeks (Figure 7B), and chondrogenesis and osteogenesis were focused on, as they are the key phenotypes for the regeneration of cartilage and bone, respectively. A marked difference in the pericyte metabolite abundance profile on stiff and rigid gels was revealed suggesting the distinct metabolic profile during the differentiation of pericytes toward two phenotypes. Furthermore, two types of lipid, the lysophosphatidic acid (LPA; such as GP18:0) in the glycerolipid pathway and the cholesterol sulfate (CS) in the steroid biosynthesis pathway were identified as key regulatory metabolites that may be involved in direct chondrogenic and osteogenic differentiation, respectively, which was further validated by their supplementation into culture medium of pericytes and MSCs on glass coverslips. These exciting findings not only confirm the intrinsic link between mechanosensing and metabolic reprogramming, but also suggest that cells may possess mechanisms to potentially translate biophysical cues to metabolic factors dictating cell fate. Notably, recent advances in metabolomics and computational analysis represents a growing trend toward the mapping of metabolism changes and the identification of key regulatory metabolites at the cell–materials interface to deepen appreciation for the role of specific metabolic factors in dictating cell fate.

## 4 Conclusions and Outlook

Recent advances in cell metabolism have refreshed our perspective of metabolism from a by‐stander to a key player. The impact of metabolic regulation on cell energy homeostasis, oxygen homeostasis and redox homeostasis, the three fundamental metabolic state, has been increasingly appreciated to actively influence cell behavior and function during differentiation, angiogenesis and immune response in the regenerative engineering scenarios. In light of the established dynamic regulatory role of biomaterial cues on cells,^2^, ^3^ the biomaterial cues that may impact cell metabolism toward modulated cell behavior have been discussed above as summarized in **Table** **3**. It seems that biomaterials not only could “give” their inherent metabolic regulatory cues (including ions, regulatory metabolites, and oxygen) to cells impacting all three aspects of metabolic state, but also could “take” cell related or derived metabolic signals (including Fe^2+^ and cell derived ROS) to indirectly modulate intracellular redox homeostasis. Through the mechanosensing network, cells are also capable of translating and transmitting certain extracellular material cues (including cell adhesivity, topography, and stiffness) into cells to regulate energy and biosynthetic homeostasis.

**Table 3:** Summary of the biomaterial‐based regulation strategies and the regulated metabolic pathways

Regulation strategy	Biomaterials examples	Metabolic pathways	Cell function	Ref.
1) Release of inherent metabolic regulator				
Ions				
	Co^2+^‐doped bioactive glass	Enhanced HIF‐1α activity (oxygen homeostasis)	Improved hMSCs survival and elevated VEGF production	76
Regulatory metabolite				
	Citrate‐based biomaterials	Facilitated the metabolic switch from glycolysis to OXPHOS leading to elevated ATP level (energy and biosynthetic homeostasis)	Promoted the phenotype progression of osteo‐differentiation with high energy demand	80
	Calcium phosphate‐bearing matrix	Elevated ATP level by providing inorganic phosphate (energy homeostasis)	Induced osteogenic differentiation	19
	Poly (Ethylene Glycol) Hydrogel containing lactate	Reducing intracellular ROS after lactate entered cells (redox homeostasis)	Improved the cell survival and function of neural pro cells	91
Oxygen	Sodium percarbonate (SPO), and PDMS–CaO_2_	Attenuated HIF‐1α accumulation under hypoxia (oxygen homeostasis)	Maintained contractility of resting skeletal muscle and under hypoxic environment	97, 100
2) Biochemical cues				
Antioxidant properties				
	poly (octamethylene citrate ascorbate) (POCA) enabling radical scavenging and iron chelation	Reduced intracellular oxidative stress (redox homeostasis)	Prolonged the viability of endothelial cells in expose to H_2_O_2_ and during rapid intracellular ROS generation	93
	Alginate/cerium oxide nanoparticles composite	Reduced intracellular oxidative stress (redox homeostasis)	protected the islet cells from oxidative damages	107
Cell adhesivity				
	Thin films of alternately layered polyelectrolytes that are biocompatible but provides poor adhesivity	Increased metabolic stress displayed as accelerated mitochondria activity (energy homeostasis)	Diffuesd organization of the actin cytoskeleton and stunted fibroblast proliferation	102
Chemical composition				
	Graphene and graphene oxide nanosheets	Disrupting mitochondria ETC and downregulating TCA cycle enzymes (energy homeostasis)	Disrupting cytoskeletal assembly and inhibitng cancner cell migration	103
3) Biophysical cues				
Surface topography				
	PDMS surface containing grooves (spacing of 1 µm, depth of 250 or 500 nm)	Enhanced ATP‐producing mitochondrial activity (energy homeostasis)	Enhanced astrocytes excitability via ATP signaling	114
	Titanium surface with micropillars (5 µm × 5 µm × 5 µm, spacing of 5 µm)	Reduced ATP and increased ROS level due to the attempted phagocytosis (energy and redox homeostasis)	Impaired osteoblast function	115
Surface stiffness				
	Collagen matrix with increasing stiffness by altering matrix density	Promoted the shift toward a more glycolytic phenotype (energy and biosynthetic homeostasis)	Increased cancer cell invasiveness	116, 117
	Self‐assembly nanofibrillar hydrogel with rigid substrate (32 KPa)	Facilitated the consumption of exogenous cholesterol sulfate for steroid biosynthesis (biosynthetic homeostasis)	Stimulated the osteogenic differentiation	113
	Self‐assembly nanofibrillar hydrogel with rigid substrate (13 KPa)	Facilitated the consumption of exogenous lysophosphatidic acid for glycerolipid biosynthesis (biosynthetic homeostasis)	Stimulated the chrondrogenic differentiaion	113

Leveraging the advances in metabolic regulation to biomaterials design could have important implications not only for cell therapy but also for advanced biomaterials design, although it is still in the emerging state and the comprehensive understanding of the mechanisms underlying the material cue‐metabolic state‐cell behavior axis presently remains preliminary. For example, citrate‐based biomaterials with their physical properties finely tuned could potentially be engineered to “give” and “take” different metabolic regulatory cues in a tunable and temporal manner to meet the dynamic need for optimal regeneration outcomes. Altogether, the integration of different metabolic pathways regulated by inherent materials cues represents a tremendous opportunity for the collaboration between cross‐disciplinary scientists and engineers to develop biomaterials, which deliberately guide cell metabolism and subsequent cell behavior for enhanced and predictable regenerative outcomes.

## Conflict of Interest

The authors declare no conflict of interest.
